# Supplementary material for: Chromothripsis during telomere crisis is independent of NHEJ, and consistent with a replicative origin
Source: Genome Res. 2019 May;29(5):737–49. doi: 10.1101/gr.240705.118 (PMC6499312; doi:10.1101/gr.240705.118)
Supplement: Supplemental Material [file supp_gr.240705.118_Supplemental_file_1.zip › contigs/annotated_contigs/DB96/contig.2.DB96_length_501_mean_cov_9.58083832335.docx]

**DB96_length_501_mean_cov_9.58083832335**

```
TACAGTCATAACAAAATAAGGTGTGCTTAAATCATCCTGCTTTTCTGGGAAATTTTTTAACATGTATACATAAATGGAGCATAAAGCCA
 >chrX:93886137-93886430 - E=8e-165
ATAAGAAACAAGGCCTGTTAATAGAAAAACCAAACTCTGTAATACATTTAAAGAGGTTTATTCCTAGCTAATATGACTGACCATGGCTC

AGGGAACAGTCTCAAGAGGCCCTGAGAAAGTGTGCCTAAATAAGTTGGCTTAGAATTTGGTTTTATACAATTTAGGAAAATGGGAATTG

TAGGTAAAATTAGAAAATTAATACAT|AGATTTATAAATATTATAT|ATTTATAATTATTTTTGTACTTTTATCTTTAAAATTGTGTAG
                                              >chrX:93857329-93857518 - E=4e-102
AGGAATAACAAGTGGAGTTATAAATAATTACAATATACTTATTTTTATATTTTTCTATGTATTACTTTTACTGGAGAACTTTCTATTTT

TTTTATGCCTTTGAGTTGCTCTCTAGTGTTTCTTATTTGATATGTAATCTGATAGAAT
```
